# The effects of probiotic supplementation on body composition, recovery following exercise‐induced muscle damage, and exercise performance: A systematic review and meta‐analysis of clinical trials

**DOI:** 10.14814/phy2.70288

**Published:** 2025-04-23

**Authors:** Nastaran Mahmoudi Shirkoohi, Hamed Mohammadi, Dler Q. Gallaly, Kurosh Djafarian

**Affiliations:** ^1^ Department of Clinical Nutrition, School of Nutritional Sciences and Dietetics Tehran University of Medical Sciences Tehran Iran; ^2^ Sports Medicine Research Center, Neuroscience Institute Tehran University of Medical Sciences Tehran Iran; ^3^ Department of Basic Sciences, College of Medicine Hawler Medical University kurdistan Iraq

**Keywords:** adults, body composition, exercise performance, muscle damage, probiotic supplementation

## Abstract

The goal of the current systematic review and meta‐analysis was to provide a definitive assessment of the impacts of probiotic supplementation on body composition, recovery from exercise‐induced muscle damage, and exercise performance in individuals actively participating in exercise. A thorough literature search encompassing Scopus, PubMed, Google Scholar, and Web of Science databases was conducted up to May 2024. The weighted mean difference (WMD) and 95% confidence interval (95% CI) for each outcome were estimated using a random‐effects model. The certainty of the assessments was further evaluated utilizing the GRADE approach. The pooled analysis showed a significant effect of probiotics on body weight [(WMD = −0.55 kg; 95% CI, −0.98 to −0.13; *p* = 0.010)], percent body fat [(WMD = −0.46%; 95% CI, −0.83 to −0.09; *p* = 0.014)], creatine kinase [(WMD = −45.57 IU. L^−1^; 95% CI: −65.12, −26.02; *p* = 0.000)], and VO_2max_ [(WMD = 1.55 mL/kg^−1^/min^−1^; 95% CI, 0.61 to 2.49; *p* = 0.001)]. Despite this, no significant effects were observed on body mass index, lean body mass, lactate dehydrogenase, and myoglobin levels. Probiotic supplementation can have significant effects on body composition and exercise performance. Due to the moderate‐to‐low certainty of evidence, further studies are warranted.

## 1 INTRODUCTION

Athletes are always looking for ways to improve their performance. Fatigue, mood disturbances, and gastrointestinal distress are the most important obstacles for athletes to achieve better performance (Clark & Mach, 2016). Furthermore, muscle discomfort caused due to exercising (EIMD) may also cause a reduction in physical activity levels and performance (Hunter et al., 2012). Dietary supplements, including probiotics, have received substantial interest in recent years for their capability effect on numerous aspects of health and overall performance, specifically in athletes (Joint, 2006).

The International Olympic Committee (IOC) introduces probiotics as useful living microorganisms for gut health and immune system modulation (Maughan et al., 2018). These nonpathogenic microorganisms can be located in fermented meal products which include yogurt, kefir, tempeh, cabbage kimchee, sauerkraut, natto, soybean‐based miso, and probiotic beverages (Calero et al., 2020). These beneficial microorganisms can help improve or replace the gut flora as well as modulate the immune system (Sánchez et al., 2017). Probiotics supplementation may increase athletic performance by positively changing the composition of gut microbiota, enhancing immune response, increasing nutrient absorption, and decreasing gastrointestinal disorders (Clark & Mach, 2016; Jäger et al., 2019; West et al., 2009). Although many studies have been conducted to investigate the effects of probiotic supplementation in adults who engage in exercise, the results are contradictory (Marttinen et al., 2020). Chen et al. have shown that mice who underwent a particular probiotic treatment (Lactobacillus plantarum at 2.05 × 10^8^ and 1.03 × 10^9^ CFU/kg/day) saw significant improvements in their physical health. Specifically, they exhibited decreased overall body weight, improved muscle mass, grip strength, and greater endurance while swimming. Additionally, following a strenuous exercise test, these mice demonstrated lowered levels of serum lactate, ammonia, creatine kinase, and glucose (Chen et al., 2016). In the case of human studies, Georges et al. indicated that 8 weeks of probiotic supplementation plus 20 g casein among healthy resistance‐trained individuals had beneficial effects on fat mass (Georges et al., 2014). However, Bifidobacterium longum's use in female swimmers for a duration of 6 weeks was unable to enhance sport performance or bolster immune function, but it did impact mood and cognitive performance (Carbuhn et al., 2018).

In alignment with the consistency observed across clinical trials, the present meta‐analysis scrutinized body composition indices, indirect indicators of muscle damage, and exercise performance in individuals actively involved in exercise, encompassing reported clinical trials (both crossover and parallel medical trials). This systematic review and meta‐analysis provide an in‐depth literature assessment focusing on the prospect that probiotic consumption may contribute to improvements in body composition, muscle damage, and exercise performance.

## 2 METHODS

### 2.1 Study protocol

This research adhered to the guidelines outlined in the Preferred Reporting Items for Systematic Reviews and Meta‐Analyses (PRISMA) statement (Moher et al., 2010) (PROSPERO registration number: CRD42024534031).

### 2.2 Search strategy

PubMed/Medline, Scopus, Web of Science, and Google Scholar were extensively searched to identify published clinical studies on probiotic supplementation and its impact on body composition, muscle damage, and exercise up to May 2024. No restrictions were imposed on language or time. The search strategy, as well as the keywords used, are elaborated in Table S1. Additionally, the “related article” function was utilized to broaden the search, and the reference lists of selected articles were scrutinized for any additional relevant studies. Duplicate citations were meticulously removed by importing all pertinent articles into EndNote software (version X9, for Windows, Thomson Reuters, Philadelphia, PA, USA).

### 2.3 Eligibility criteria

The titles and abstracts of all identified articles were reviewed for eligibility. To meet the inclusion criteria, studies had to adhere to the following PICOS standards Table S2 (Nang et al., 2015). All trials included in the current meta‐analysis shared the following characteristics: (1) clinical trials with parallel or crossover designs, (2) participants aged 18 and over, (3) participants receiving some form of probiotic supplementation as a nutritional strategy, (4) outcomes including at least one of the specified factors in this study, (5) provision of adequate information to calculate effect sizes for the outcome measures. Studies conducted on children or adolescents, animals, in vitro or in vivo experiments were excluded, in addition to cross‐sectional studies, controlled studies, reviews, experiments, and observations. Furthermore, studies using probiotics (vitamins, minerals, etc.) solely in the intervention group without a control group were also excluded.

### 2.4 Selection strategy

After the initial screening process, all articles retrieved from online databases or manual searches were imported into Endnote software (EndNote X9, Thomson Reuters, New York) for further analysis. Two independent reviewers (N.M. and H.M.) reviewed the items for inclusion and exclusion, selecting relevant sentences for a comprehensive manuscript review. Clinical studies meeting the inclusion criteria were all included in this meta‐analysis. The selection of studies for review was based on a standardized form utilizing data from the complete articles.

### 2.5 Data extraction

The following information was extracted from the full texts of the included studies using a predefined data abstraction form: study details (first author's name, study location, publication year, study design, and total sample size), participant characteristics (gender, age, sport type, training status, and BMI), intervention details (probiotic species, dosage, administration method, and duration), control group regimens, measurement timing, and mean changes with standard deviations for all outcomes mentioned across the trial duration for both intervention and control groups. Additionally, Plot Digitizer (http://plotdigitizer.sourceforge.net/) was utilized to extract numerical estimates from graphs. Any disagreements during the study selection process and data extraction were resolved via a face‐to‐face discussion with a third reviewer (K.J).

### 2.6 Risk of bias assessment

We evaluated the qualitative information based on the Cochrane Risk of Bias Assessment Tool II (Sterne et al., 2019). Two independent investigators assessed the quality of the studies in five key domains, which included the risk of bias related to randomization, interventions, missing outcome data, outcome measurement, and selection of reported results. For each domain, terms such as “Low,” “Some concerns,” or “High” were used to make evaluations.

### 2.7 Data synthesis and statistical analysis

The meta‐analysis included mean changes and their standard deviations (SDs) of variables related to body composition, muscle damage, and performance post‐probiotic supplementation compared to a control group. While the results were initially presented as standard errors (SEs), 95% confidence intervals (CIs), and interquartile ranges (IQRs), they were also converted to SDs utilizing the method proposed by Hozo et al. (2005). A random effects model (Dersimonian‐Liard) was used to calculate the combined weighted mean difference (WMD) to account for all available differences (DerSimonian & Laird, 1986). Significant heterogeneity was considered present if *I* ^2^ exceeded 50% or if *p* was less than 0.05 following a visual assessment of forest plots or Cochrane's Q test (Higgins & Thompson, 2002). To pinpoint the source of heterogeneity, predefined subgroup analyses were conducted based on the intervention duration (≤6 vs. >6 weeks), intervention species (single vs. multi‐strain), training status (trained vs. untrained), age (≤30 years vs. 30 > years), sex (male, female, and both), baseline BMI (18.5–24.9, 25–29.9, ≥30), and study design (parallel vs. crossover).

For sensitivity analysis, we systematically removed each study individually and reevaluated the pooled assessments to gauge the impact. Publication bias in reporting the studies was evaluated using both the funnel plot and the Egger test (Vandenbroucke, 1998). The meta‐analysis was carried out utilizing STATA R version 17.0 software by Stata Corp, based in College Station, Lakeway, TX, USA.

### 2.8 Certainty assessment

The quality of evidence was evaluated the use of the GRADE (Grading of recommendations assessment, development, and evaluation) guideline. evidence is categorized into four categories: high, moderate, low, or very low (Guyatt et al., 2008).

## 3 RESULTS

### 3.1 Study selection

**FIGURE 1:**
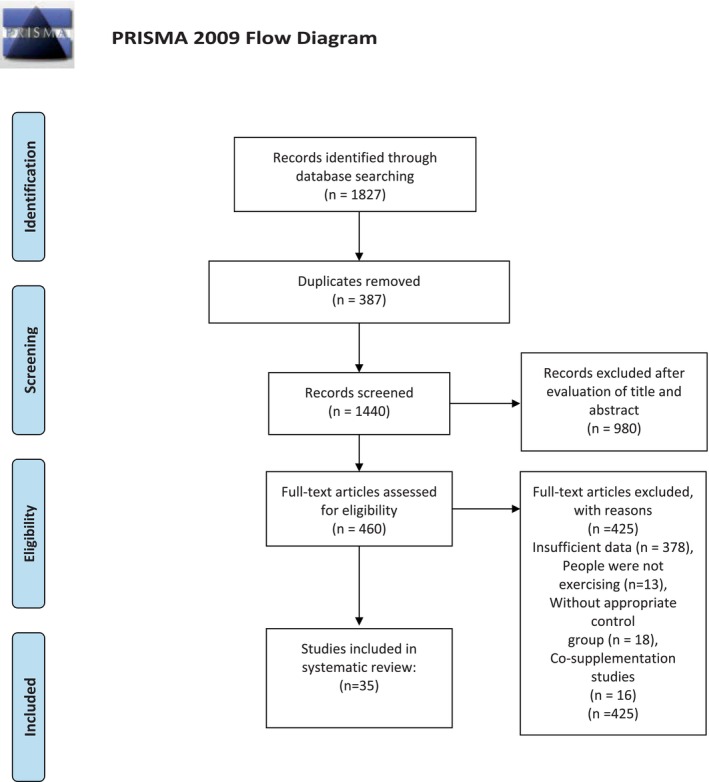
The Preferred Reporting Items for Systematic Review and Meta‐analysis (PRISMA) flowchart.

In our systematic search, we initially identified 1827 papers across various databases. Among these, 387 were identified as duplicates, and 980 were deemed irrelevant based on screening their titles and abstracts. Following a thorough examination of the full texts, a total of 35 out of 460 studies (including 41 effect sizes) met the criteria for quantitative evaluation regarding the impact of probiotic supplementation on body composition, muscle damage, and exercise outcomes. The visual representation depicting the study selection process is available in Figure 1.

### 3.2 Characteristics of the included studies

In the present meta‐analysis, a total of 1336 participants were included, with 656 participants in the control group and 680 in the intervention group. The following nations participated in the studies: Poland (Mazur‐Kurach et al., 2022; Przewlocka et al., 2023; Smarkusz‐Zarzecka et al., 2020), Taiwan (Cheng et al., 2023; Fu et al., 2021; Huang et al., 2018; Huang, Lee, et al., 2019; Huang, Wei, et al., 2019; Lee, Ho, et al., 2022; Lee, Jhang, et al., 2021; Lee, Liao, et al., 2022; Lin et al., 2020), USA (Jäger, Purpura, et al., 2016; Jäger, Shields, et al., 2016; Jose Antonio et al., 2018; Toohey et al., 2020; Townsend et al., 2018; Zabriskie et al., 2020), Israel (Hoffman et al., 2019; Schreiber et al., 2021), Japan (Inoue et al., 2018; Komano et al., 2018; Sawada et al., 2019), Korea (Lim et al., 2020; Sohn et al., 2021), Malaysia (Ibrahim et al., 2018; Salleh et al., 2021), Canada (Smith‐Ryan et al., 2019), China (Li et al., 2023; Wang et al., 2024), UK (Marshall et al., 2017), Sweden (Axling et al., 2020), Slovakia (Hric et al., 2021), Australia (Cox et al., 2010), and Iran (Hajipoor et al., 2021) from 2008 to 2024. In regard to the selected studies, the characteristics and details were showcased in Table S3. All included studies were clinical trials with parallel design, except for six that were crossover (Cox et al., 2010; Fu et al., 2021; Jäger, Purpura, et al., 2016; Jäger, Shields, et al., 2016; Lee, Jhang, et al., 2021; Zabriskie et al., 2020). During the follow‐up period ranging from 2 to 16 weeks, participants' mean age varied from 19 to 70 years, with baseline BMI values ranging from 18.9 to 35.45 kg/m^2^. Some studies enrolled only men (Cheng et al., 2023; Cox et al., 2010; Hoffman et al., 2019; Huang et al., 2018; Huang, Wei, et al., 2019; Ibrahim et al., 2018; Jäger, Purpura, et al., 2016; Jäger, Shields, et al., 2016; Komano et al., 2018; Lee, Jhang, et al., 2021; Li et al., 2023; Mazur‐Kurach et al., 2022; Przewlocka et al., 2023; Sawada et al., 2019; Schreiber et al., 2021; Townsend et al., 2018) or women (Axling et al., 2020; Hric et al., 2021; Salleh et al., 2021; Smith‐Ryan et al., 2019; Toohey et al., 2020), while other studies included both genders (Fu et al., 2021; Hajipoor et al., 2021; Huang, Lee, et al., 2019; Inoue et al., 2018; Jose Antonio et al., 2018; Lee, Ho, et al., 2022; Lee, Liao, et al., 2022; Lim et al., 2020; Lin et al., 2020; Marshall et al., 2017; Smarkusz‐Zarzecka et al., 2020; Sohn et al., 2021; Wang et al., 2024; Zabriskie et al., 2020). Furthermore, the clinical trials included individuals with different training statuses. Specifically, 20 studies involved trained participants (Axling et al., 2020; Cox et al., 2010; Huang, Lee, et al., 2019; Huang, Wei, et al., 2019; Inoue et al., 2018; Jäger, Purpura, et al., 2016; Jäger, Shields, et al., 2016; Jose Antonio et al., 2018; Komano et al., 2018; Li et al., 2023; Lin et al., 2020; Marshall et al., 2017; Mazur‐Kurach et al., 2022; Przewlocka et al., 2023; Salleh et al., 2021; Sawada et al., 2019; Schreiber et al., 2021; Smarkusz‐Zarzecka et al., 2020; Toohey et al., 2020; Townsend et al., 2018) while 15 studies enrolled untrained individuals (Cheng et al., 2023; Fu et al., 2021; Hajipoor et al., 2021; Hoffman et al., 2019; Hric et al., 2021; Huang et al., 2018; Ibrahim et al., 2018; Lee, Ho, et al., 2022; Lee, Jhang, et al., 2021; Lee, Liao, et al., 2022; Lim et al., 2020; Smith‐Ryan et al., 2019; Sohn et al., 2021; Wang et al., 2024; Zabriskie et al., 2020). Moreover, most studies used single strain for intervention (Axling et al., 2020; Cheng et al., 2023; Cox et al., 2010; Fu et al., 2021; Hoffman et al., 2019; Huang et al., 2018; Huang, Lee, et al., 2019; Huang, Wei, et al., 2019; Jäger, Shields, et al., 2016; Komano et al., 2018; Lee, Ho, et al., 2022; Lee, Liao, et al., 2022; Li et al., 2023; Lim et al., 2020; Lin et al., 2020; Salleh et al., 2021; Sawada et al., 2019; Sohn et al., 2021; Toohey et al., 2020; Townsend et al., 2018; Zabriskie et al., 2020) and 14 studies used multi‐strain (Hajipoor et al., 2021; Hric et al., 2021; Ibrahim et al., 2018; Inoue et al., 2018; Jäger, Purpura, et al., 2016; Jose Antonio et al., 2018; Lee, Jhang, et al., 2021; Marshall et al., 2017; Mazur‐Kurach et al., 2022; Przewlocka et al., 2023; Schreiber et al., 2021; Smarkusz‐Zarzecka et al., 2020; Smith‐Ryan et al., 2019; Wang et al., 2024).

Some of the articles conducted various follow‐up assessments, such as evaluating muscle damage indices for both the intervention and placebo groups immediately, within 3 h, as well as 24‐, 48‐, and 72‐h post‐exercise. Fourteen trials in 12 studies stated immediate follow‐up (Cheng et al., 2023; Fu et al., 2021; Hoffman et al., 2019; Huang et al., 2018; Huang, Wei, et al., 2019; Jäger, Shields, et al., 2016; Komano et al., 2018; Lee, Jhang, et al., 2021; Lee, Liao, et al., 2022; Lin et al., 2020; Sawada et al., 2019; Zabriskie et al., 2020). Ten trials in eight studies had ≤3 h. follow‐up times (Cheng et al., 2023; Fu et al., 2021; Huang, Wei, et al., 2019; Jäger, Purpura, et al., 2016; Lee, Ho, et al., 2022; Lee, Jhang, et al., 2021; Lee, Liao, et al., 2022; Zabriskie et al., 2020). Five trials from four studies documented 24‐h follow‐up assessments (Fu et al., 2021; Jäger, Purpura, et al., 2016; Lee, Ho, et al., 2022; Przewlocka et al., 2023), while four trials across three studies conducted follow‐ups 48 h post‐exercise (Fu et al., 2021; Jäger, Purpura, et al., 2016; Lee, Ho, et al., 2022), and three trials from three studies carried out follow‐ups 72 h after exercise (Fu et al., 2021; Jäger, Purpura, et al., 2016; Zabriskie et al., 2020).

For the investigation conducted by Huang, Lee, et al. (2019) that considered two different doses (3 and 9 × 10^10^ CFU), the investigation conducted by Mon‐chien Lee et al. and Lee et al. that used a heated kill and live strain (Lee, Ho, et al., 2022; Lee, Liao, et al., 2022), the investigation conducted by Huang, Wei, et al. (2019) performed on two different triathlon teams, the investigation conducted by Smarkusz‐Zarzecka et al. (2020) performed on male and female, and the investigation conducted by Hajipoor et al. (2021) used supplements with or without vitamin D, we consider two arms for all aforementioned studies.

### 3.3 Meta‐analysis findings

#### 3.3.1 Effects of probiotic supplementation on BMI

**FIGURE 2:**
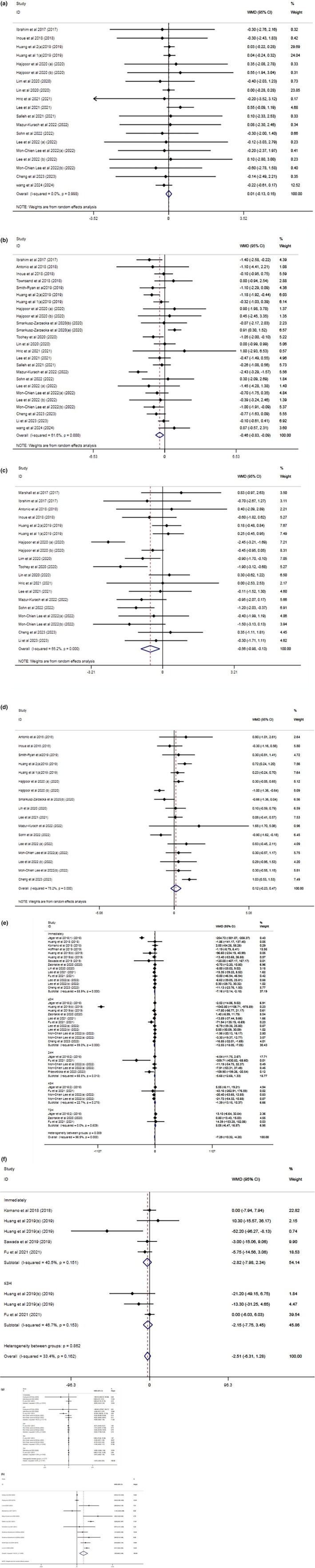
Forest plot detailing weighted mean difference and 95% confidence intervals (CIs) for the effect of probiotics supplementation on (a) BMI (kg/m^2^); (b) body weight (kg); (c) %BF (%); (d) LBM (kg); (e) CK (IU. L^−1^); (f) LDH (IU. L^−1^); (g) Mb (mg/dL), and (h) VO_2max_ (mL/kg/min).

BMI was reported as an outcome measure in 15 studies with 710 participants (intervention = 372 and control = 338) and 19 effect sizes. The random‐effects model's overall findings revealed that probiotic supplementation did not influence BMI (WMD: 0.009 kg/m^2^, 95% CI: −0.13, 0.14, *p* = 0.899). No significant heterogeneity was found between studies (*I* ^2^ = 0.0%, *p* = 0.998) (Figure 2a).

#### 3.3.2 Effect of probiotic supplementation on BW

The impact of probiotic supplementation on body weight was evaluated in 16 studies encompassing 19 effect sizes. The combined outcomes from the random‐effects model unveiled a substantial alteration in body weight post‐probiotic supplementation (WMD: −0.55 kg, 95% CI: −0.98 to −0.13; *p* = 0.010) (Figure 2b). Notably, a moderate level of heterogeneity was observed across the studies (*I* ^2^ = 65.2%, *p* < 0.001). Subsequent subgroup analyses indicated a significant reduction in body weight post‐probiotic supplementation in trials lasting more than 6 weeks, studies incorporating females and both genders, trials conducted on participants over the age of 30 who were classified as overweight or obese, and experiments involving untrained individuals (Table S4).

#### 3.3.3 Effect of probiotic supplementation on PBF

Figure 2c presents the pooled effect of probiotic supplementation on PBF in 20 studies with 25 effect sizes. A significant reduction of PBF after consumption of probiotics was noted (WMD: −0.46; 95% CI, −0.83 to −0.09; *p* = 0.014). Likewise, between‐study heterogeneity was noted (*I* ^2^ = 61.6%, *p* < 0.001). Moreover, an evaluation of the results from subgroup analysis indicated that probiotic supplementation in individuals aged ≤30 with a typical baseline BMI, along with studies lasting fewer than 6 weeks and employing a single‐strain probiotic, resulted in a notable reduction in PBF (Table S4).

#### 3.3.4 Effect of probiotic supplementation on LBM

In the context of our investigation involving 13 studies and 17 arms of clinical trials (Figure 2d), the impact of probiotic supplementation on lean body mass (LBM) was found to be statistically insignificant (WMD: 0.11 kg; 95% CI: −0.23, 0.47; *p* = 0.508). Notably, there was substantial variation observed across the studies. Moreover, remarkable heterogeneity was observed between studies (*p* = <0.001, *I* ^2^ = 78.2%). Furthermore, the subgroup analysis indicated a noteworthy enhancement in LBM specifically among male and trained participants (Table S4).

#### 3.3.5 Effect of probiotic supplementation on CK

Following the analysis of 37 trials, it was found that probiotic supplementation exhibited a remarkable reduction effect on creatine kinase (CK) concentration overall (WMD = −47.57 IU.L^−1^; 95% CI: −65.12, −26.02; *p* = 0.000). This was accompanied by a high level of heterogeneity (*p* < 0.001, *I* ^2^ = 96.9%) (Figure 2e). Subgroup analyses were carried out to assess whether the impact of probiotic supplementation on serum CK levels varied based on post‐exercise follow‐up periods, gender, probiotic strains, training status, and baseline body mass index (BMI) (Table S4). Multiple studies have demonstrated that probiotic supplementation led to decreased CK concentrations in males with normal BMI levels, particularly in trials where CK was evaluated immediately and 3 h post‐exercise, as well as studies involving single‐strain probiotics.

#### 3.3.6 Effect of probiotic supplementation on LDH

Upon conducting a meta‐analysis comprising four trials encompassing 8 effect sizes, it was found that there were no notable alterations in serum LDH concentrations in the probiotic supplementation group when compared to the control group (WMD = −3.62 IU. L^−1^; 95% CI: −9.01, 1.76; *p* = 0.188). Also, nonsignificant heterogeneity was observed among the articles (*p* = 0.162, *I* ^2^ = 33.4%) (Figure 2f) (Table S4).

#### 3.3.7 Effect of probiotic supplementation on MB

A comprehensive analysis of 16 effect sizes of 170 participants found no significant change in MB content in individuals in the probiotic supplement group compared with the control group (WMD = −1.96 mg/dL; 95% CI, −3.94 to −0.22; *p* = 0.009). Furthermore, no significant heterogeneity was observed between products (*p* = 0.707, *I* ^2^ = 0.0%) (Figure 2g) (Table S4).

#### 3.3.8 Effect of probiotic supplementation on VO_2max_

Pooling together 11 effect sizes from 10 studies revealed a beneficial impact of probiotic supplementation on maximal oxygen consumption (VO_2max_) in the intervention group compared to the placebo (WMD = 1.55 mL/kg/min; 95% CI, 0.61–2.49; *p* = 0.001). There was also significant heterogeneity between studies (*I* ^2^ = 93.2%, *p* < 0.001). Subgroup analysis was performed to reduce the potential for heterogeneity. These observations suggest that similar results were obtained in trials with trained participants and follow‐up periods of less than 6 weeks (Figure 2f) (Table S4).

### 3.4 Sensitivity analysis

Sensitivity analysis findings concerning myoglobin (MB) revealed a noteworthy impact on the overall results following the exclusion of studies carried out by Mon‐Chien Lee et al. 2021; Lee, Jhang, et al. (2021) (WMD = −1.56 mg/dL, 95% CI: −3.51 to 0.37), Zabriskie et al. (2020) (WMD = −1.60 mg/dL, 95% CI: −3.24 to 0.03), and Fu et al. (2021) (WMD = −2.00 mg/dL, 95% CI: −3.99 to −0.007).

### 3.5 Publication bias

**FIGURE 3:**
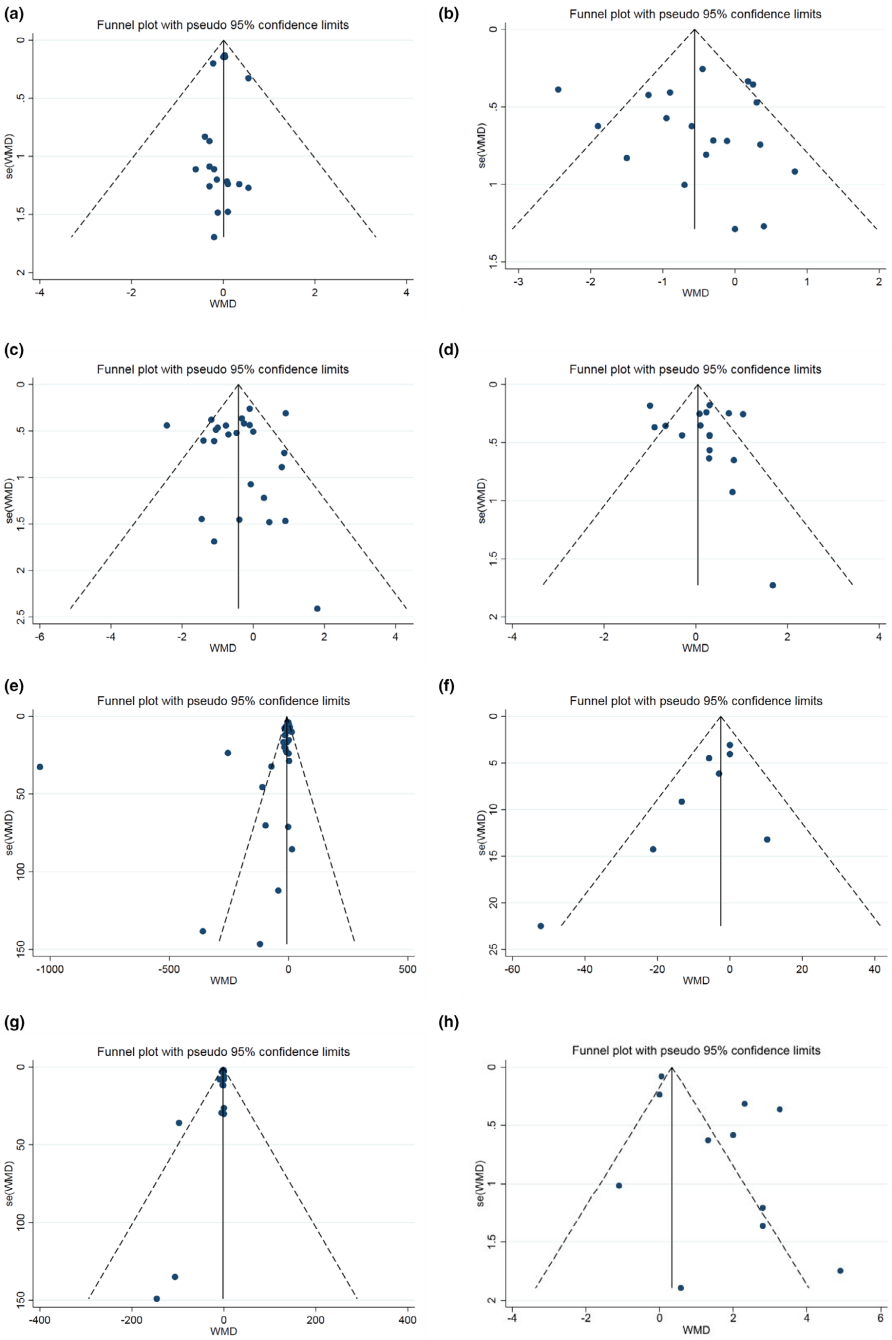
Funnel plots for the effect of probiotics supplementation on (a) BMI (kg/m^2^); (b) body weight (kg); (c) %BF (%); (d) LBM (kg); (e) CK (IU. L^−1^); (f) LDH (IU. L^−1^); (g) Mb (mg/dL), and (h) VO_2max_ (mL/kg/min).

According to Begg's test, there was no indication of publication bias for variables such as lean body mass (*p* = 0.86), percent body fat (*p* = 0.51), body mass index (*p* = 0.46), body weight (*p* = 0.75), and maximal oxygen consumption (VO_2max_) (*p* = 0.81). However, signs of publication bias were detected for creatine kinase (CK) (*p* = 0.00), lactate dehydrogenase (LDH) (*p* = 0.02), and myoglobin (MB) (*p* = 0.04). Subsequently, Egger's regression test revealed no significant publication bias for LDH (*p* = 0.07), lean body mass (*p* = 0.73), percent body fat (*p* = 0.91), body mass index (*p* = 0.68), body weight (*p* = 0.88), and maximal oxygen consumption (VO_2max_) (*p* = 0.051), whereas significant publication bias was noted for CK (*p* = 0.04) and MB (*p* = 0.04). The observed funnel plots also corroborated these results (Figure 3a–h).

### 3.6 Risk of bias and GRADE assessment

In the risk of bias assessment for the 35 eligible studies provided in Table S5, it was found that 18 trials exhibited a low risk of bias across all domains (Fu et al., 2021; Hoffman et al., 2019; Huang et al., 2018; Inoue et al., 2018; Lee, Jhang, et al., 2021; Lin et al., 2020; Mazur‐Kurach et al., 2022; Przewlocka et al., 2023; Salleh et al., 2021; Sawada et al., 2019; Schreiber et al., 2021; Smarkusz‐Zarzecka et al., 2020; Smith‐Ryan et al., 2019; Sohn et al., 2021; Toohey et al., 2020; Townsend et al., 2018; Wang et al., 2024; Zabriskie et al., 2020), while 12 trials raised some concerns about bias (Axling et al., 2020; Cheng et al., 2023; Hajipoor et al., 2021; Huang, Lee, et al., 2019; Huang, Wei, et al., 2019; Ibrahim et al., 2018; Jäger, Purpura, et al., 2016; Jäger, Shields, et al., 2016; Lee, Ho, et al., 2022; Lee, Liao, et al., 2022; Li et al., 2023; Lim et al., 2020), and five trials presented a high risk of bias (Cox et al., 2010; Hric et al., 2021; Jose Antonio et al., 2018; Komano et al., 2018; Marshall et al., 2017). The certainty of evidence was assessed using the GRADE protocol for measured outcomes outlined in Table S6. The evidence quality for variables such as body weight and PBF was considered high due to low‐to‐moderate bias risk, low heterogeneity, and narrow confidence intervals. However, the evidence related to BMI, LDH, and VO_2max_ was of moderate quality due to significant imprecision and inconsistency (*I* ^2^ = 93.2%) in the case of VO_2max_. On the other hand, the evidence for LBM, CK, and MB was downgraded to low quality due to serious inconsistency for LBM (*I* ^2^ = 78.2%) and CK (*I* ^2^ = 96.9%), as well as serious imprecision (wide CI) for LBM and MB, and significant publication bias (*p* = 0.04) for both CK and MB. If you have any questions about the quality assessment or would like to discuss the implications of bias on study outcomes, feel free to ask. I am here to provide further insights and explanations on the assessment of study quality and evidence certainty.

## 4 DISCUSSION

This study is the first systematic review and meta‐analysis to explore the relationship between Probiotic supplementation and body composition, recovery post‐exercise‐induced muscle damage, and exercise performance. The combined findings from 35 eligible studies involving 1336 participants suggest that probiotic supplementation resulted in decreased body weight (BW), percentage body fat (PBF), creatine kinase (CK) levels, and improved maximal oxygen consumption (VO_2max_) compared to the control group. However, there was no significant effect on body mass index (BMI), lean body mass (LBM), lactate dehydrogenase (LDH), and myoglobin (MB) levels. Subgroup analysis revealed that probiotic use for over 6 weeks in participants over 30 years old who were overweight or obese had a positive impact on body weight. Additionally, the use of single‐strain probiotics in males with a normal BMI post‐exercise showed a decrease in creatine kinase levels. Interestingly, taking probiotics for less than 6 weeks in trained individuals improved performance by enhancing VO_2max_.

The exploration of probiotics in the field of sports sciences has been relatively limited concerning potential applications and advantages. In a systematic review conducted by Di Dio et al. (2023) the inclusion of specific bacterial strains in athletes' diets and the consumption of multi‐strain compounds have shown promise in enhancing performance and positively affecting factors such as fatigue, muscle soreness, body composition, and cardiorespiratory fitness. Similarly, a thorough review and meta‐analysis of 24 studies demonstrated that probiotic supplementation was associated with increased overall muscle strength and muscle mass, although it did not exhibit a significant effect on total lean body mass (Prokopidis et al., 2023). Within a recent systematic review and meta‐analysis, Antibañez‐Gutierrez et al. highlighted the potential advantages of probiotic supplementation in enhancing performance in a test focused on aerobic metabolism among trained individuals. Our study findings align with this assertion, demonstrating a significant reduction in body weight and body fat percentage, alongside enhancements in VO_2max_, while observing no substantial effects on lean body mass (LBM), lactate dehydrogenase (LDH), and body mass index (BMI) when comparing probiotic supplementation to the control group (Santibañez‐Gutierrez et al., 2022). In contrast to our study's findings, Coqueiro et al. (2017) reported that probiotic supplementation does not exhibit an ergogenic effect, partly attributed to the limited number of studies available. Apart from human research, the beneficial impact of probiotic supplementation has been illustrated in animal studies focusing on anthropometric measurements and muscle damage indicators. Following a 4‐week regimen of Lactobacillus plantarum PL‐02 supplementation and resistance exercise training, mice displayed notable improvements, such as increased maximum crawl count, enhanced exercise endurance, reduced climbing time, and decreased post‐exercise levels of lactate, blood ammonia, CK, and blood urea nitrogen (BUN) (Yeh et al., 2022). Additionally, research by Lee, Hsu, et al. (2021) indicated that supplementing with Lactobacillus plantarum for 4 weeks significantly boosted muscle mass, strength, endurance, and glycogen storage in mice's muscles and liver. Furthermore, Lactobacillus plantarum contributed to considerable reductions in post‐exercise levels of CK, lactate, BUN, and ammonia. Recent studies have highlighted the significant role of intestinal microorganisms and reduced gut inflammation in the development of obesity and related conditions (Cani & Jordan, 2018). Probiotics play a crucial role in regulating gut microbiota balance by inhibiting harmful bacteria growth, suppressing the production of detrimental metabolic byproducts and pro‐inflammatory cytokines, enhancing carbohydrate metabolism, and modulating the immune system (Gérard, 2016; Kober & Bowe, 2015; La Fata et al., 2018). Consequently, probiotics hold promise in preventing and managing obesity stemming from disrupted gut bacteria balance and chronic inflammatory bowel conditions. EIMD is a common occurrence among individuals undertaking intense physical activities (Clarkson & Sayers, 1999). This damage triggers a cascade of physiological responses in the body, including inflammation, muscle discomfort, increased intramuscular proteins (such as CK, LDH, and Mb), swelling, and diminished muscle functionality (Hunt et al., 2021). CK elevation post‐exercise acts as an indicator of skeletal muscle tissue harm (Willoughby et al., 2003). The precise mechanisms through which bacterial supplementation influences muscle damage remain incompletely understood. The potential elevation of short‐chain fatty acids (SCFA) in the gut due to probiotics could be a contributing factor. This rise in SCFA levels might aid in raising glycogen levels and enhancing protein absorption, potentially promoting muscle recovery and repair (Giron et al., 2022). Vigorous physical exercise often leads to reactive oxygen species (ROS) production, which can contribute to muscle damage and decreased performance (Alessio, 2006; Giron et al., 2022). Lactic acid bacteria have been shown to possess antioxidant properties such as scavenging oxidizing compounds, inhibiting intestinal ROS generation, and regulating the expression of nuclear factor erythrocyte 2‐related factor 2 (Nrf2) to alleviate oxidative stress and inflammation (Ng et al., 2009; Plaza‐Diaz et al., 2014). Furthermore, by stimulating SCFA production, these bacteria can help reduce circulating endotoxins, inflammation, and oxidative stress (Amaretti et al., 2013; Iddir et al., 2020; Wang et al., 2017). Nevertheless, further research is necessary to fully comprehend the intricate mechanisms influenced by probiotics, including the application of methodologies such as proteomics and metabolomics. It is noteworthy to consider the impact of various probiotic strains on athletes' performance across different sports. Probiotics have the potential to enhance exercise capacity by bolstering the host's immune system function, intestinal barrier integrity, energy metabolism, mental resilience, and antioxidant capability. These benefits are often attributed to probiotics' ability to metabolize carbohydrates into SCFAs like acetic acid, butyric acid, and propionic acid (Zhang et al., 2023). Additionally, Scheiman et al. (2019) discovered that Veillonella organisms could enhance endurance performance by 13% through a metabolic advantage achieved by the colonization of lactate‐metabolizing microorganisms converting it into propionate. Recognizing the profound potential of this finding, researchers are exploring the development of probiotic capsules containing Veillonella to elevate athletes' performance through increased species presence in their gut microbiota. Furthermore, evidence supports that when probiotics are combined with a fiber‐rich diet, recovery time post‐intense training periods can be reduced, alleviating gastrointestinal issues, psychological stress, oxidative stress, and other concerns associated with overtraining, while also preventing upper respiratory tract infections. These combined benefits have been demonstrated to enhance sports performance (Meeusen et al., 2013; Pane et al., 2018). In clinical settings, regular intake of specific probiotic strains may bolster immune function and diminish the duration of illness experienced by athletes during training or competitions. Certain probiotic strains have displayed efficacy in reducing the severity of respiratory infections and gastrointestinal disturbances as well. The advantages of probiotics hinge on the specific strain and dosage employed, potentially including augmented gut‐barrier function, nutrient assimilation, and improvements in athletic recovery and performance.

### 4.1 Strengths and limitations

Numerous strengths merit consideration in this investigation. Firstly, it is imperative to acknowledge that this study stands out as one of the initial comprehensive meta‐analyses delving into the impacts of probiotic supplementation on body composition, muscle injury, and exercise performance. The second notable strength of this current meta‐analysis lies in its detailed assessment of <24‐, 24‐, 48‐, and 72‐h post‐exercise follow‐up measurements of muscle damage indicators, accompanied by subgroup analyses based on follow‐up timelines, gender, age, initial BMI, probiotic strains, and intervention durations, illustrating the influence of probiotics on body composition, muscle injury, and exercise performance. Thirdly, a substantial volume of trials has been undertaken on individuals actively involved in exercise. Furthermore, we employed rigorous GRADE assessment methodologies, sensitivity evaluations, and subgroup analyses to gauge study quality, identify publication biases, and pinpoint potential sources of heterogeneity across the majority of trials, respectively.

This meta‐analysis encountered several limitations, notably the substantial heterogeneity present among the studies included. However, a random effects model was employed in an effort to reduce the impact of this heterogeneity on the estimated effect sizes. (1) The microbiome of these athletes has never been studied before. Therefore, it is impossible to determine which strain or strains may be changed, eliminated, or enhanced by probiotic treatment. Understanding this microbiome is essential in order to comprehend the alterations induced by the administration of various probiotic strains and the impact of sports activity. (2) It is important to recognize that there may be other variables, such as diet, medication usage, especially antibiotics, and other supplements, that could potentially influence body composition, muscle damage, and performance. These variables were not accounted for in the study.

## 5 CONCLUSION

This meta‐analysis, characterized by low to very low levels of evidence certainty, indicated notable impacts of probiotic supplementation on body composition, exercise damage recovery, and exercise performance. Nonetheless, given the restricted certainty of the evidence, additional extensive and meticulously designed trials are necessary to substantiate these conclusions.

## AUTHOR CONTRIBUTIONS

Nastaran Mahmoudi Shirkoohi was involved in data curation (equal), formal analysis (equal), investigation (equal), and writing‐original draft (equal). Hamed Mohammadi was involved in conceptualization (equal), methodology (lead), writing‐review and editing (lead). Dler Q. Gallaly was involved in resources (lead), validation (lead), and visualization (lead). Kurosh Djafarian was involved in project administration (equal), supervision (equal), writing‐review and editing (lead).

## CONFLICT OF INTEREST STATEMENT

The authors have no conflict of interest to declare.

## Supporting information


Table S1.

Table S2.

Table S3.

Table S4.

Table S5.

Table S6.
