# Supplementary material for: The effects of probiotic supplementation on body composition, recovery following exercise‐induced muscle damage, and exercise performance: A systematic review and meta‐analysis of clinical trials
Source: Physiol Rep. 2025 Apr 23;13(8):e70288. doi: 10.14814/phy2.70288 (PMC12018167; doi:10.14814/phy2.70288)
Supplement: Supplementary file 1 — Table S1. [file PHY2-13-e70288-s001.docx]

**Table1.** Search terms used across the various databases.

| **PubMed: 608** | **“(((((("Probiotics"[Mesh]) AND ((((((((((((((((((((((((((((((((((((("Body Composition"[Mesh]) OR "Body Weight"[Mesh]) OR "Body Mass Index"[Mesh]) OR "Creatine Kinase"[Mesh]) OR "L-Lactate Dehydrogenase"[Mesh]) OR "Myoglobin"[Mesh]) OR "Athletic Injuries"[Mesh]) OR "Athletic Performance"[Mesh]) OR (Body Composition[Title/Abstract])) OR (Body Weight[Title/Abstract])) OR (Body Mass Index[Title/Abstract])) OR (BMI[Title/Abstract])) OR (Creatine Kinase[Title/Abstract])) OR (L-Lactate Dehydrogenase[Title/Abstract])) OR (LDH[Title/Abstract])) OR (Myoglobin[Title/Abstract])) OR (Athletic Injuries[Title/Abstract])) OR (Athletic Performance[Title/Abstract])) OR (VO2MAX[Title/Abstract]))”** |
| --- | --- |
| **Web of science: 239** | **https://www.webofscience.com/wos/woscc/summary/d3e2fdb4-0386-429d-a07e-c447d347f5ee-c5837678/relevance/1** |
| **scupos:980** | **( ( ( TITLE-ABS-KEY ( probiotic) ) ) AND ( ( TITLE-ABS-KEY ( "Body Composition" ) OR TITLE-ABS-KEY ( "Body Weight" ) OR TITLE-ABS-KEY ( "Body Mass Index" ) TITLE-ABS-KEY ( "Creatine Kinase" ) OR TITLE-ABS-KEY ( "L-Lactate Dehydrogenase" ) OR TITLE-ABS-KEY ( myoglobin ) ( "Athletic Injuries" ) OR TITLE-ABS-KEY ( "Athletic Performance" ) ) ) ) AND ( ( TITLE-ABS-KEY ( "clinical trial" ) OR TITLE-ABS-KEY ( "clinical trial" ) OR TITLE-ABS-KEY ( "randomized clinical trial" ) OR TITLE-ABS-KEY ( rct ) OR TITLE-ABS-KEY ( "interventional trial" ) OR TITLE-ABS-KEY ( "Creatine Kinase" ) OR TITLE-ABS-KEY ( "double blind clinical trial" ) OR TITLE-ABS-KEY ( "triple blind clinical trial" ) OR TITLE-ABS-KEY ( "randomized double blind clinical trial" ) ) )** |
