# Supplementary material for: The effects of probiotic supplementation on body composition, recovery following exercise‐induced muscle damage, and exercise performance: A systematic review and meta‐analysis of clinical trials
Source: Physiol Rep. 2025 Apr 23;13(8):e70288. doi: 10.14814/phy2.70288 (PMC12018167; doi:10.14814/phy2.70288)
Supplement: Supplementary file 2 — Table S2. [file PHY2-13-e70288-s006.docx]

**Table 2.** PICOS criteria for inclusion of studies

| Parameter Inclusion Criteria | Parameter Inclusion Criteria |
| --- | --- |
| Population | Both trained and untrained adults engaged in exercise |
| Intervention | Administration of probiotics either from food or supplements in any form, including capsule, tablet, and powder at any duration |
| Control | Comparison with placebo, or any pharmacological or non-pharmacological intervention(s) |
| Outcome | Those which reported mean changes and their standard deviations (SDs) of BMI, BW, PBF, LBM, CK, LDH, Mb, VO2_max_ throughout the trial for both intervention and control groups or presented required information for calculation of those effect sizes |
| Study design | Parallel and crossover clinical trials |

**Abbreviations:** SDs, standard deviations; BMI, body mass index; BW, body weight; PBF, percent body fat; LBM, lean body mass; CK, creatine kinase; LDH, lactate dehydrogenase; Mb, myoglobin; Vo2_max,_ maximal oxygen consumption
