# Supplementary material for: The effects of probiotic supplementation on body composition, recovery following exercise‐induced muscle damage, and exercise performance: A systematic review and meta‐analysis of clinical trials
Source: Physiol Rep. 2025 Apr 23;13(8):e70288. doi: 10.14814/phy2.70288 (PMC12018167; doi:10.14814/phy2.70288)
Supplement: Supplementary file 3 — Table S3. [file PHY2-13-e70288-s003.docx]

Table 3: Characteristics of the studies

| Studies | Country | Study Design | Participant | Sex | Sample size | | Trial Duration  (week) | Mean age | Mean  BMI | Intervention | | | | Outcomes |
| --- | --- | --- | --- | --- | --- | --- | --- | --- | --- | --- | --- | --- | --- | --- |
|  |  |  |  |  | **IG** | **CG** |  |  |  | **Species of probiotic** | **Dose (CFU)** | **Control** | **Frequency**  **and type** |  |
| **Wang et al 2024** | China | Parallel,  R, PC, DB | Marathon runners | B | 10 | 9 | 5 | 29.14 | 21.02 | Lactobacillus acidophilus, Bifidobacterium longum | 6.8 × 10^10^ CFU Lactobacillus acidophilus  and 3.3 × 10^10^ CFU of Bifidobacterium longum | Placebo (maltodextrin) | NR | BMI, PBF |
| **Przewłócka et al 2023** | Poland | Parallel,  R, PC, DB | MMA athletes | M | 11 | 12 | 4 | 25.45 | 24.78 | Bifidobacterium lactis, Levilacto bacillus brevis, Lactobacillus acidophilus, Bifidobacterium bifidum and Lactococcus lactis | 2.5×10^9^ | Placebo (40 mg of maltodextrin and plant proteins) | Caps  2#BID | CK |
| **Cheng et al 2023** | Taiwan | Parallel,  R, PC, DB | Healthy adults | M | 15 | 15 | 6 | 20.7 | 23.69 | Heat-killed Lactiplantibacillus plantarum | 3×10^10^ | Placebo | Caps  2#BID | CK, BW, BMI, PBF, LBM |
| **Li et al 2023** | China | Parallel,  R, PC, SB | cross-country skiing | M | 7 | 8 | 8 | 19.45 | 18.9 | Bifidobacterium lactis | 1×10^9^ | Placebo (yogurt) | Caps  4#QID  (3 with meals and 1 before sleep) | BW, PBF, VO2_max_ |
| **Mon-Chien Lee et al 2022 (a)** | Taiwan | Parallel,  R, PC, DB | Untrained subjects | B | 35 | 35 | 6 | 21.7 | 22.95 | Live Lactobacillus paracasei | 2×10^10^ | Placebo (micro crystalline cellulose) | Caps  2#BID | CK, MB, BW, BMI, PBF, LBM |
| **Mon-Chien Lee et al 2022 (b)** | Taiwan | Parallel,  R, PC, DB | Untrained subjects | B | 35 | 35 | 6 | 21.7 | 22.95 | Heat-killed Lactiplantibacillus paracasei | 2×10^10^ | Placebo (micro crystalline cellulose) | Caps  2#BID | CK, MB, BW, BMI, PBF, LBM |
| **Lee et al 2022 (a**) | Taiwan | Parallel,  R, PC, DB | Healthy subjects | B | 8 | 9 | 6 | 22.2 | 24.25 | Viable Lactiplantibacillus plantarum | 3×10^11^ | Placebo (maltodextrin and micro crystalline cellulose) | Caps  3#TDS | CK, BMI, PBF, LBM |
| **Lee et al 2022 (b**) | Taiwan | Parallel,  R, PC, DB | Healthy subjects | B | 9 | 9 | 6 | 22.5 | 23.35 | Heat-killed Lactiplantibacillus plantarum | 3×10^11^ | Placebo (maltodextrin and micro crystalline cellulose) | Caps  3#TDS | CK, BMI, PBF, LBM |
| **Sohn et al 2022** | South Korea | Parallel,  R, PC, DB | Obese adults | B | 41 | 40 | 12 | 46.65 | 27.2 | Lactobacillus plantarum | 4×10^9^ | Placebo | Caps  1#QD | BMI, BW, PBF, LBM |
| **Mazur-Kurach et al 2022** | Poland | Parallel,  R, PC, DB | Road cyclists | M | 13 | 13 | 16 | 22.26 | 21.29 | Lactobacillus plantarum, Lactobacillus casei, Lactobacillus rhamnosus, Bifidobacterium breve, Lactobacillus acidophilus, Bifidobacterium longum, Bifidobacterium bifidum, Bifidobacterium infantis, Lactobacillus helveticus, Lactobacillus fermentum, Lactobacillus bulgaricus, Lactococcus lactis, and Streptococcus thermophilus | 1×10^11^ | Placebo (potato starch) | Caps  1#QD | BMI, BW, PBF, LBM, VO2_max_ |
| **Fu et al 2021** | Taiwan | Cross-over,  R, PC, DB | Recreational runners | B | 8 | 8 | 4 | 25.5 | 23 | Lactobacillus plantarum | 3×10^10^ | Placebo | Caps  2#BID | CK, LDH, MB |
| **Lee et al 2021** | Taiwan | Cross over, DB | Healthy  and untrained males | M | 16 | 16 | 4 | 25.1 | 23.83 | SYNKEFIR (Lactobacillus paracasei, Lactobacillus casei, Lactobacillus kefiranofaciens, and Lactococcus lactis) | 20g | Placebo | 1 pouch | CK, LDH, BMI, BW, PBF, LBM, |
| **Salleh et al 2021** | Malaysia | Parallel,  R, PC, DB | Badminton Players | F | 15 | 15 | 6 | 19.7 | 23.45 | Lactobacillus casei Shirota | 3×10^10^ | Placebo | Drink with 120 ml orange juice | BMI, PBF, VO2_max_ |
| **Hric et al 2021** | Slovakia | Parallel,  R, DB | Healthy females | F | 13 | 9 | 4 | 47.5 | 25.4 | Bryndza cheese (Lactococcus, Streptococcus, Lactobacillus and enterococcus) | 30g | Without the regular consumption of Bryndza cheese | Daily | BMI, BW, PBF |
| **Schreiber et al 2021** | Israel | Parallel,  R, PC, DB | Elite cyclists | M | 11 | 16 | 12 | 28.3 | 23.2 | Lactobacillus helveticus Lafti, Bifidobacterium animalis ssp. lactis Lafti, Enterococcus faecium Bifidobacterium longum, Bacillus subtilis | 15 ×10^9^ | Placebo (potato starch, magnesium stearate, ascorbic acid and white vegetable powder) | Caps  1#QD | Vo2_max_ |
| **Zabriskie et al 2020** | USA | Cross over,  R, PC, DB | Healthy, active subjects | B | 15 | 16 | 2 | 29.9 | 25.49 | Saccharomyces cerevisiae | 250mg | Placebo (maltodextrin) | Daily (before and 72h post exercise) | CK, MB |
| **Lin et al 2020** | Taiwan | Parallel,  R, PC, DB | Middle And Long-Distance Runners | B | 11 | 10 | 5 | 21.4 | 19.85 | Bifidobacterium longum | 1.5×10^10^ | Placebo | Caps  3#TDS | CK, BMI, BW, PBF, LBM |
| **Toohey et al 2020** | USA | Parallel,  R, PC, DB | Division I female athletes | F | 11 | 12 | 10 | 19.6 | 23.35 | Bacillus subtilis | 5 × 10^9^ | Placebo | Caps  1#QD | BW, PBF |
| **Axling et al 2020** | Sweden | Parallel,  R, PC, DB | Healthy non-anemic athlete | F | 14 | 14 | 12 | 21.95 | 23.1 | Lactobacillus plantarum and 20 mg of iron | 10^10^ | Iron, maize starch, maltodextrin, cellulose derivatives, magnesium stearate | Caps  1#QD | VO2_max_ |
| **Smarkusz-Zarzecka 2020 (a)** | Poland | Parallel,  R, PC, DB | Long-Distance Runners | F | 14 | 6 | 12 | 39.73 | 38.22 | Bifidobacterium lactis, Lactobacillus brevis, Lactobacillus casei, Lactococcus lactis, Lactobacillus acidophilus, Bifidobacterium bifidum and Lactobacillus salivarius | 2.5×10^9^ | Placebo | Caps  2#BID | PBF, VO2_max_ |
| **Smarkusz-Zarzecka 2020 (b)** | Poland | Parallel,  R, PC, DB | Long-Distance Runners | M | 20 | 26 | 12 | 35.27 | 34.02 | Bifidobacterium lactis, Lactobacillus brevis, Lactobacillus casei, Lactococcus lactis, Lactobacillus acidophilus, Bifidobacterium bifidum and Lactobacillus salivarius | 2.5×10^9^ | Placebo | Caps  2#BID | PBF, LBM, VO2_max_ |
| **Hajipoor et al 2020 (a)** | Iran | Parallel,  R, PC, DB | Obese adults | B | 28 | 31 | 10 | 38.13 | 35.45 | Multistrain (Lactobacillus Acidophilus and Bifidobacterium lactis) | 4 × 10^7^ | Plain low-fat yogurt | 100 grams per day | BMI, BW, PBF, LBM |
| **Hajipoor et al 2020 (b)** | Iran | Parallel,  R, PC, DB | Obese adults | B | 29 | 30 | 10 | 42.35 | 34.35 | Multistrain (Lactobacillus Acidophilus and Bifidobacterium lactis) +1,000 IU vitamin D | 4 × 10^7^ | Vitamin D-fortified yogurt | 100 grams per day | BMI, BW, PBF, LBM |
| **Lim et al 2020** | Korea | Parallel,  R, PC, DB | Overweight subjects | B | 47 | 48 | 12 | 46.8 | 28.4 | Lactobacillus sakei | 5×10^9^ | Placebo | Daily | BMI, BW |
| **Sawda et al 2019** | Japan | Parallel,  R, PC, DB | Long distance relay race participants | M | 24 | 25 | 12 | 19.95 | 19.3 | Lactobacillus gasseri | 1×10^10^ | Placebo | 200 ml of beverages | CK, LDH |
| **Hoffman et al 2019** | Israel | Parallel,  R, PC, DB | Soldiers | M | 8 | 8 | 2 | 20.1 | 23.39 | Bacillus coagulans | 1×10 ^9^ | Placebo | 1 serving per day with breakfast | CK |
| **Huang et al 2019 (a)** | Taiwan | Parallel, DB | Sprint triathlon teams | M | 9 | 9 | 4 | 20.65 | 22.3 | Lactobacillus plantarum | 1.5×10^10^ | Placebo  (Microcrystalline cellulose) | Caps  2 #BID  after training and before sleeping | CK, LDH |
| **Huang et al 2019 (b)** | Taiwan | Parallel, DB | Championship  triathlon teams | M | 9 | 9 | 3 | 21.2 | 22.4 | Lactobacillus plantarum | 1.5×10^10^ | Placebo  (Microcrystalline cellulose) | Caps  2 #BID after training and before sleeping | CK, LDH, MB |
| **Huang et al 2019 (a)** | Taiwan | Parallel,  R, PC, DB | Healthy participants | B | 18 | 18 | 6 | 21.8 | 22.2 | Lactobacillus plantarum | 3×10^10^ | Placebo (maltodextrin and micro crystalline cellulose | Caps  3#TDS | BMI, BW, PBF, LBM |
| **Huang et al 2019 (b)** | Taiwan | Parallel,  R, PC, DB | Healthy participants | B | 18 | 18 | 6 | 22.05 | 22.22 | Lactobacillus plantarum | 9×10^10^ | Placebo (maltodextrin and micro crystalline cellulose | Caps  3#TDS | BMI, BW, PBF, LBM |
| **Smith Ryan et al 2019** | Canada | Parallel,  R, PC, DB | Healthcare workers | F | 15 | 18 | 6 | 30.35 | 24.7 | Multistrain (Bifidobacterium bifidum. Bifidobacterium lactis, Bifidobacterium lactis, Lactobacillus acidophilus, Lactobacillus brevis, Lactobacillus casei, Lactobacillus salivarius, and Lactococcus lactis) | 2.5×10^9^ | Placebo (maize starch and maltodextrin) | Sachet prior to the first meal of the day | PBF, LBM, VO2_max_ |
| **Komano et al 2018** | Japan | Parallel,  R, PC, DB | Track and field, futsal, and football player | M | 26 | 24 | 2 | 20.65 | 20.65 | Lactococcus lactis | 1×10^11^ | Placebo (cornstarch) | Caps  1#QD | CK, LDH |
| **Huang et al 2018** | Taiwan | Parallel,  R, PC, DB Trial | Healthy participants | M | 8 | 8 | 6 | 20-30 | 24.99 | Lactobacillus plantarum | 1×10^11^ | Placebo | Caps  1#QD | Vo2_max_ |
| **Inoue et al 2018** | Japan | Parallel,  R, PC, DB | Healthy elderly | B | 20 | 18 | 12 | 70.3 | 23.5 | Multiple Bifidobacterium | 1.25×10^10^ | Placebo | Sachet after breakfast | BMI, BW, PBF, LBM |
| **Townsend et al 2018** | USA | Parallel,  R, PC, DB | Baseball athletes | M | 13 | 12 | 12 | 20.1 | 25.29 | Bacillissubtilis | 1×10^9^ | Placebo  (maltodextrin) | Caps  1#QD | PBF |
| **Antonio et al 2018** | USA | Parallel,  R, PC, DB | Aerobic and/or resistance trainer | B | 10 | 10 | 6 | 27.5 | 24.39 | Multi strain (Bifidobacterium and Streptococcus thermophilus) | 1×10^10^ | Placebo (maltodextrin) | Caps  1#QD | BW, PBF, LBM |
| **Ibrahim et al 2017** | Malaysia | Parallel,  R, PC, DB | Circuit trainer | M | 9 | 12 | 12 | 21.5 | 21.6 | Lactobacillus acidophilus, Lactobacillus casei, Lactobacillus lactis, Lactobacillus bifidum, Lactobacillus infantis and Lactobacillus longum | 3×10^10^ | Placebo | Sachets  2#BID | BMI, BW, PBF |
| **Marshall et al 2017** | UK | Parallel,  R, PC | Marathon competitors | B | 11 | 13 | 12 | 41 | 25.12 | Multi strain (Lactobacillus acidophilus, Lactobacillus acidophilus, Lactobacillus acidophilus CUL-21 Bifidobacterium bifidum and lactis, Bifidobacterium bifidum CUL-20 and Bifidobacterium animalis subspecies lactis CUL-34 | 1×10^10^ | Maintenance of regular diet | Caps  1#QD | BW, VO2_max_ |
| **Jager et al 2016** | USA | Cross over, SB/Placebo and diet controlled | Recreationally-trainer | M | 29 | 29 | 2 | 21.5 | 28.85 | Bacillus coagulans GBI-30,6086 plus 20g casein protein | 1 ×10^9^ | 20g casein protein | Mixture of probiotic and 20g casein protein daily | CK |
| **Jager et al 2016** | USA | Cross over,  PC, DB | Resistance-trainer | M | 15 | 15 | 3 | 25 | NR | Streptococcus thermophilus and Bifidobacterium breve | 5 ×10^9^ afu | Placebo | Caps  1#QD | CK |
| **Cox et al 2008** | Australia | Cross over,  R, PC, DB | Highly-trained distance runners | M | 20 | 20 | 4 | 27.3 | 20.58 | Lactobacillus fermentum | 1.2× 10^10^ | Placebo | Caps  3#TDS | Vo2_max_ |

Abbreviations: IG: Intervention Group; CG: Control Group; R: Randomized; DB: Double Blind; SB: Single Blind; PC: Placebo-Control; CFU: Colony-Forming Unit, M: Male; F: Female; B: Both; AFU: Active-Forming Unit, QD: Once a day, BID: Twice a day, TDS: Three times a day, CK: Creatine kinase, LDH: Lactate Dehydrogenase, MB: Myoglobin, BW: Body Weight, BMI: Body Mass Index, PBF: Percent Body Fat, LBM: Lean Body Mass; VO2_max_: Maximal Oxygen Consumption
