# Supplementary material for: The effects of probiotic supplementation on body composition, recovery following exercise‐induced muscle damage, and exercise performance: A systematic review and meta‐analysis of clinical trials
Source: Physiol Rep. 2025 Apr 23;13(8):e70288. doi: 10.14814/phy2.70288 (PMC12018167; doi:10.14814/phy2.70288)
Supplement: Supplementary file 4 — Table S4. [file PHY2-13-e70288-s002.docx]

**Table** 4. Subgroup analyses of probiotic supplementation on on body composition, recovery following exercise induced muscle damage, and exercise performance

|  | NO | WMD (95%CI) | P within group | Heterogeneity | | |
| --- | --- | --- | --- | --- | --- | --- |
|  |  |  |  | P heterogeneity | I^2^ | P between sub-groups |
| Subgroup analyses of probiotic supplementation on BMI (kg/m^2^) | | | | | | |
| Overall effect | 19 | -0.011 (-0.11, 0.13) | 0.870 | 0.997 | 0.0% |  |
| Subgroup analyses of probiotic supplementation on Body Weight (kg) | | | | | | |
| Overall effect | 19 | -0.56 (-0.98, -0.14) | **0.009** | <0.001 | 68.5% |  |
| Gender | | | | | | |
| Male | 5 | -0.38 (-1.01, 0.24) | 0.229 | 0.698 | 0.0% | 0.168 |
| Female | 2 | -1.53 (-2.64, -0.43) | **0.006** | 0.184 | 43.2% |  |
| Both | 12 | -0.47 (-0.71, -0.24) | **0.000** | <0.001 | 77.8% |  |
| Age (years) | | | | | | |
| ≤ 30 | 12 | -0.15 (-0.44, 0.12) | 0.276 | 0.041 | 45.9% | 0.000 |
| > 30 | 7 | -0.94 (-1.26, -0.62) | **0.000** | <0.001 | 74.7% |  |
| Baseline BMI (kg/m^2^) | | | | | | |
| Normal (18.5-24.9) | 13 | -0.18 (-0.45, 0.09) | 0.201 | 0.053 | 42.4% | 0.001 |
| Overweight (25-29.9) | 4 | -0.83 (-1.36, -0.29) | **0.002** | 0.213 | 33.1% |  |
| Obese (≥30) | 2 | -1.05 (-1.47, -0.63) | **0.000** | <0.001 | 94.6% |  |
| Training status | | | | | | |
| Trained | 9 | -0.05 (-0.36, 0.25) | 0.728 | 0.051 | 48.1% | 0.000 |
| Untrained | 10 | -0.91 (-1.20, -0.62) | **0.000** | 0.002 | 65.3% |  |
| Probiotic strain | | | | | | |
| Single-strain | 10 | -0.30 (-0.57, -0.03) | **0.026** | 0.002 | 65.6% | 0.019 |
| Multi-strain | 9 | -0.82 (-1.17, -0.48) | **0.000** | <0.001 | 68.6% |  |
| Trial duration (week) | | | | | | |
| ≤ 6 | 9 | 0.03 (-0.27, 0.35) | 0.805 | 0.360 | 9.1% | 0.000 |
| > 6 | 10 | -0.97 (-1.26, -0.68) | **0.000** | 0.002 | 65.9% |  |
| Subgroup analyses of probiotic supplementation on PBF (%) | | | | | | |
| Overall effect | 25 | -0.47 (-0.83, -0.12) | **0.008** | <0.001 | 64.2% |  |
| Gender | | | | | | |
| Male | 7 | -0.30 (-0.60, -0.004) | **0.047** | <0.001 | 86.8% | 0.335 |
| Female | 5 | -0.63 (-1.16, -0.10) | **0.019** | 0.490 | 0.0% |  |
| Both | 13 | -0.57 (-0.83, -0.31) | **0.000** | 0.193 | 24.8% |  |
| Age (years) | | | | | | |
| ≤ 30 | 17 | -0.66 (-0.86, -0.45) | **0.000** | 0.001 | 59.2% | 0.000 |
| > 30 | 8 | 0.33 (-0.09, 0.76) | 0.122 | 0.153 | 34.5% |  |
| Baseline BMI (kg/m^2^) | | | | | | |
| Normal (18.5-24.9) | 18 | -0.66 (-0.86, -0.46) | **0.000** | 0.002 | 56.1% | 0.000 |
| Overweight (25-29.9) | 3 | 0.72 (-0.62, 2.07) | 0.294 | 0.849 | 0.0% |  |
| Obese (≥30) | 2 | 0.67 (-1.36, 2.71) | 0.516 | 0.829 | 0.0% |  |
| Training status | | | | | | |
| Trained | 12 | -0.37 (-0.59, -0.15) | **0.001** | <0.001 | 79.3% | 0.103 |
| Untrained | 13 | -0.70 (-1.03, -0.37) | **0.000** | 0.510 | 0.0% |  |
| Probiotic strain | | | | | | |
| Single-strain | 13 | -0.55 (-0.77, -0.33) | **0.000** | 0.159 | 28.4% | 0.199 |
| Multi-strain | 12 | -0.29 (-0.62, 0.04) | 0.086 | <0.001 | 77.4% |  |
| Trial duration(week) | | | | | | |
| ≤ 6 | 14 | -0.63 (-0.87, -0.39) | **0.000** | 0.305 | 13.6% | 0.044 |
| > 6 | 11 | -0.25 (-0.53, 0.03) | 0.089 | <0.001 | 79.2% |  |
| Subgroup analyses of probiotic supplementation on LBM (kg) | | | | | | |
| Overall effect | 17 | 0.12 (-0.21, 0.45) | 0.486 | <0.001 | 79.5% |  |
| Gender | | | | | | |
| Male | 3 | 0.56 (0.21, 0.90) | **0.002** | 0.024 | 73.2% | 0.007 |
| Female | 2 | -0.38 (-0.97, 0.20) | 0.197 | 0.150 | 51.8% |  |
| Both | 12 | 0.04 (-0.11, 0.19) | 0.607 | <0.001 | 81.2% |  |
| Age (years) | | | | | | |
| ≤ 30 | 11 | 0.44 (0.26, 0.61) | **0.000** | 0.219 | 23.6% | 0.000 |
| > 30 | 6 | -0.38 (-0.60, -0.17) | **0.000** | <0.001 | 83.6% |  |
| Baseline BMI (kg/m^2^) | | | | | | |
| Normal (18.5-24.9) | 13 | 0.40 (0.23, 0.58) | **0.000** | 0.197 | 24.4% | 0.000 |
| Obese (≥30) | 2 | -0.33 (-0.58, -0.08) | **0.008** | <0.001 | 96.2% |  |
| Training status | | | | | | |
| Trained | 7 | 0.26 (0.04, 0.49) | **0.017** | 0.019 | 60.3% | 0.053 |
| Untrained | 10 | -0.008 (-0.18, 0.16) | 0.927 | <0.001 | 84.8% |  |
| Probiotic strain | | | | | | |
| Single-strain | 9 | 0.39 (0.20, 0.58) | **0.000** | 0.003 | 65.9% | 0.000 |
| Multi-strain | 8 | -0.25 (-0.45, -0.04) | **0.015** | <0.001 | 78.9% |  |
| Trial duration(week) | | | | | | |
| ≤ 6 | 11 | 0.43 (0.25, 0.61) | **0.000** | 0.245 | 20.8% | 0.000 |
| > 6 | 6 | -0.40 (-0.62, -0.19) | **0.000** | 0.000 | 83.5% |  |
| Subgroup analyses of probiotic supplementation on CK concentration (IU.L^-1^) | | | | | | |
| (Follow-ups after exercise  Immediate | 14 | -25.05 (-44.88, -5.22) | **0.013** | <0.001 | 88.9% |  |
| ≤3h | 11 | -98.59 (-152.52, -44.65) | **0.000** | <0.001 | 99.0% |  |
| 24h | 5 | -19.22 (-47.32, 8.86) | 0.180 | 0.017 | 66.7% |  |
| 48h | 4 | -10.03 (-28.12, 8.05) | 0.277 | 0.110 | 50.2% |  |
| 72h | 3 | 5.05 (-6.46, 16.56) | 0.390 | 0.609 | 0.0% |  |
| Gender | | | | | | |
| Male | 18 | -9.78 (-13.60, -5.97) | **0.000** | <0.001 | 98.5% | 0.046 |
| Both | 19 | -3.73 (-8.28, 0.80) | 0.107 | 0.466 | 0.0% |  |
| Baseline BMI (kg/m^2^) | | | | | | |
| Normal (18.5-24.9) | 29 | -11.67 (-15.75, -7.60) | **0.000** | <0.001 | 97.3% | 0.007 |
| Overweight (25-29.9) | 4 | -4.81 (-11.51, 1.89) | 0.160 | <0.001 | 97.4% |  |
| Training status | | | | | | |
| Trained | 13 | -11.92 ( -16.88, -6.95) | **0.000** | <0.001 | 98.9% | 0.023 |
| Untrained | 24 | -4.82 (-8.43, -1.21) | **0.009** | 0.432 | 2.1% |  |
| Probiotic strain | | | | | | |
| Single-strain | 30 | -9.45 (-13.01, -5.88) | **0.000** | <0.001 | 97.5% | 0.037 |
| Multi-strain | 7 | -2.83 (-7.93, -2.27) | 0.277 | 0.072 | 48.2% |  |
| Subgroup analyses of probiotic supplementation on LDH concentration (IU.L^-1^) | | | | | | |
| (Follow-ups after exercise  Immediate | 5 | -3.24 (-10.92, 4.43) | 0.408 | 0.151 | 40.5% |  |
| ≤3h | 3 | -6.82 (-19.08, 5.43) | 0.275 | 0.153 | 46.7% |  |
| Subgroup analyses of probiotic supplementation on Myoglobin concentration (mg/dL) | | | | | | |
| (Follow-ups after exercise  Immediate | 3 | -0.04 (-5.22, 5.13) | 0.986 | 0.618 | 0.0% |  |
| ≤3h | 5 | -13.44 (-38.45, 11.55) | 0.292 | 0.114 | 46.3% |  |
| 24h | 3 | -4.74 (-13.91, 4.43) | 0.311 | 0.768 | 0.0% |  |
| 48h | 3 | -1.46 (-3.33, 0.41) | 0.126 | 0.725 | 0.0% |  |
| 72h | 2 | -3.61 (-9.00, 1.78) | 0.190 | 0.492 | 0.0% |  |
| Subgroup analyses of probiotic supplementation on Vo_2max_ (mL/kg/min) | | | | | | |
| Overall effect | 11 | 1.55 (0.61, 2.49) | **0.001** | <0.001 | 93.2% |  |
| Gender | | | | | | |
| Male | 6 | 0.95 (0.60, 1.29) | **0.000** | <0.001 | 88.2% | 0.000 |
| Female | 4 | 0.23 (0.08, 0.38) | **0.002** | <0.001 | 96.6% |  |
| Age (years) | | | | | | |
| ≤ 30 | 7 | 0.30 (0.16, 0.44) | **0.000** | <0.001 | 95.4% | 0.005 |
| > 30 | 4 | 1.38 (0.64, 2.12) | **0.000** | 0.042 | 63.3% |  |
| Training status | | | | | | |
| Trained | 9 | 0.34 (0.20, 0.49) | **0.000** | <0.001 | 94.1% | 0.782 |
| Untrained | 2 | 0.28 (-0.14, 0.71) | 0.197 | <0.001 | 90.1% |  |
| Probiotic strain | | | | | | |
| Single-strain | 5 | 0.29 (0.15, 0.43) | **0.000** | <0.001 | 96.8% | 0.001 |
| Multi-strain | 6 | 1.50 (0.79, 2.21) | **0.000** | 0.031 | 59.4% |  |
| Trial duration(week) | | | | | | |
| ≤ 6 | 5 | 1.42 (1.11, 1.73) | **0.000** | <0.001 | 94.5% | 0.000 |
| > 6 | 6 | 0.08 (-0.07, 0.23) | 0.291 | 0.004 | 70.6% |  |

Abbreviations: CI, confidence interval; WMD, weighted mean differences; BMI, body mass index; BW, body weight; PBF, percent body fat; LBM, lean body mass; CK, creatine kinase; LDH, lactate dehydrogenase; Mb, myoglobin; Vo2_max,_ maximal oxygen consumption
