# Supplementary material for: The effects of probiotic supplementation on body composition, recovery following exercise‐induced muscle damage, and exercise performance: A systematic review and meta‐analysis of clinical trials
Source: Physiol Rep. 2025 Apr 23;13(8):e70288. doi: 10.14814/phy2.70288 (PMC12018167; doi:10.14814/phy2.70288)
Supplement: Supplementary file 5 — Table S5. [file PHY2-13-e70288-s004.docx]

**Table 5. Risk of bias assessment**

|  | **Randomization process** | **Deviations from the intended interventions** | **Missing outcome data** | **Measurement of outcome** | **Selection of the reported result** | **General risk of bias** |
| --- | --- | --- | --- | --- | --- | --- |
| Wang et al 2024 | L | L | L | L | L | L |
| Przewłócka et al 2023 | L | L | L | L | L | L |
| Cheng et al 2023 | S.C | L | L | L | L | S.C |
| Li et al 2023 | L | S.C | L | S.C | L | S.C |
| Mon-Chien Lee et al 2022 | S.C | L | L | L | L | S.C |
| Lee et al 2022 | S.C | L | L | L | L | S.C |
| Mazur-Kurach et al 2022 | L | L | L | L | L | L |
| Sohn et al 2022 | L | L | L | L | L | L |
| Fu et al 2021 | L | L | L | L | L | L |
| Lee et al 2021 | L | L | L | L | L | L |
| Salleh et al 2021 | L | L | L | L | L | L |
| Hric et al 2021 | S.C | S.C | S.C | L | H | H |
| Schreiber et al 2016 | L | L | L | L | L | L |
| Zabriskie et al 2020 | L | L | L | L | L | L |
| Lin et al 2020 | L | L | L | L | L | L |
| Toohey et al 2020 | L | L | L | L | L | L |
| Axling et al 2020 | L | L | S.C | L | L | S.C |
| Smarkusz-Zarzecka et al 2020 | L | L | L | L | L | L |
| Hajipoor et al 2020 | L | L | S.C | L | L | S.C |
| Lim et al 2020 | S.C | L | S.C | L | L | S.C |
| Sawda et al 2019 | L | L | L | L | L | L |
| Hoffman et al 2019 | L | L | L | L | L | L |
| Huang et al 2019 (1) | S.C | L | S.C | L | L | S.C |
| Huang et al a 2019 (2) | S.C | L | S.C | L | L | S.C |
| Smith-Ryan et al 2019 | L | L | L | L | L | L |
| Komano et al 2018 | L | L | L | H | L | H |
| Huang et al 2018 | L | L | L | L | L | L |
| Inoue et al 2018 | L | L | L | L | L | L |
| Townsend et al 2018 | L | L | L | L | L | L |
| Antonio et al 2018 | S.C | L | L | L | H | H |
| Ibrahim et al 2017 | L | S.C | L | L | L | S.C |
| Marshall et al 2017 | S.C | S.C | L | L | H | H |
| Jäger et al 2016(1) | L | S.C | L | S.C | L | S.C |
| Jäger et al 2016(2) | S.C | L | L | L | L | S.C |
| cox et al 2008 | L | L | L | L | H | H |

Abbreviations: L, Low-risk of bias; H, High-risk of bias; S.C, Some Concern about risk of bias
