# Supplementary material for: The effects of probiotic supplementation on body composition, recovery following exercise‐induced muscle damage, and exercise performance: A systematic review and meta‐analysis of clinical trials
Source: Physiol Rep. 2025 Apr 23;13(8):e70288. doi: 10.14814/phy2.70288 (PMC12018167; doi:10.14814/phy2.70288)
Supplement: Supplementary file 6 — Table S6. [file PHY2-13-e70288-s005.docx]

**Table 6.** GRADE profile of probiotic supplementation on body composition, recovery following exercise-induced muscle damage, and performance.

| Outcomes | Risk of bias | Inconsistency | Indirectness | Imprecision | Publication Bias | Number  of intervention/control | Quality  of evidence |
| --- | --- | --- | --- | --- | --- | --- | --- |
| BMI | No Serious limitation | No Serious limitation | No Serious limitation | Serious limitation^2^ | No serious limitation | 710  (372/338) | ⊕⊕⨁◯  moderate |
| BW | No serious limitation | No Serious limitation | No Serious limitation | No serious limitation | No serious limitation | 709 (361/348) | ⊕⊕⊕⊕  high |
| PBF | No serious limitation | No Serious limitation | No Serious limitation | No Serious limitation | No serious limitation | 867 (451/416) | ⊕⊕⊕⊕  high |
| LBM | No serious limitation | Serious limitation^1^ | No Serious limitation | Serious limitation^2^ | No serious limitation | 666  (353/313) | ⊕⊕◯◯  Low |
| CK | No serious limitation | Serious limitation^1^ | No Serious limitation | No Serious limitation | Serious limitation^3^ | 566  (291/275) | ⊕⊕◯ ◯  Low |
| LDH | No serious limitation | No Serious limitation | No Serious limitation | Serious limitation^2^ | No serious limitation | 135 (68/67) | ⊕⊕⨁◯  moderate |
| MB | No serious limitation | No Serious limitation | No Serious limitation | Serious limitation^2^ | Serious limitation^3^ | 170 (84/86) | ⊕⊕◯ ◯  Low |
| Vo_2max_ | No serious limitation | Serious limitation^1^ | No Serious limitation | No Serious limitation | No serious limitation | 305 (148/157) | ⊕⊕⨁◯  moderate |

| 1. There is high heterogeneity for LBM (I2=78.2%), CK (I2=96.9), VO2max (I2=93.2). 2. There is no evidence of significant effects of probiotic supplementation on BMI, LBM, LDH and MB. 3. There is evidence of publication bias for CK (P=0.04) and MB (P=0.04). 4. ⊕ = serious limitation; ◯ = no serious limitation |
| --- |
